# Anthropometric indicators in traditional peoples and communities in Brazil: analysis of individual records from the Food and Nutrition Surveillance System, 2019

**DOI:** 10.1590/S2237-96222023000400005.EN

**Published:** 2023-12-18

**Authors:** Italo Wesley Oliveira Aguiar, Antônio Augusto Ferreira Carioca, Brena Barreto Barbosa, Lia Silveira Adriano, Anael Queirós Silva Barros, Carl Kendall, Ligia Regina Franco Sansigolo Kerr

**Affiliations:** 1 Universidade Federal do Ceará, Programa de Pós-Graduação em Saúde Pública, Fortaleza, CE, Brazil; 2 Universidade de Fortaleza, Pós-Graduação em Saúde Coletiva, Fortaleza, CE, Brazil; 3 Universidade Estadual do Ceará, Programa de Pós-Graduação em Nutrição e Saúde, Fortaleza, CE, Brazil; 4 Universidade de Fortaleza, Curso de Nutrição, Fortaleza, CE, Brazil; 5 Universidade Estadual do Ceará, Programa de Pós-Graduação em Saúde Coletiva, Fortaleza, CE, Brazil; 6 Tulane University, Department of Global Community Health and Behavioral Sciences, New Orleans, LA, United States

**Keywords:** Nutritional Epidemiology, Primary Health Care, Health Information Systems, Quilombolas, Roma (Ethnic Group), Descriptive Studies, Epidemiología Nutricional, Primeros Auxilios, Sistemas de Información en Salud, Quilombolas, Romaní, Estudios Descriptivos, Epidemiologia Nutricional, Atenção Primária à Saúde, Sistemas de Informação em Saúde, Quilombolas, Roma (Grupo Étnico), Estudos Descritivos

## Abstract

**Objective:**

To describe the prevalence of underweight and obesity indicators among individuals registered as traditional peoples and communities in the Food and Nutrition Surveillance System, across Brazil, in 2019.

**Methods:**

This was a descriptive study using individual secondary data from participants receiving care in the Primary Health Care within the Brazilian National Health System.

**Results:**

In the study population (N = 13,944), there was a higher prevalence of short stature among male children and adolescents (14.2%), when compared to their female counterparts (11.8%); in the adult female population, there was a higher prevalence of obesity (23.0%), when compared to the male population (11.3%); the prevalence of low height-for-age in riverine communities (18.5%) and obesity in the adult faxinalense population (75.1%) stood out.

**Conclusion:**

Anthropometric disparities between different communities require tailored responses, emphasizing targeted primary health care and programs to ensure food and nutrition security.

## Study contributions

**Main results:**

It was possible to identify a higher prevalence of low weight and height-for-age in the child population of riverine communities, while faxinalense communities showed a higher prevalence of obesity in adults.

**Implications for services:**

Continuous improvement in the implementation of SISVAN protocols is recommended, as well as strengthening the assistance provided to communities with the worst indicators.

**Perspectives:**

Studies assessing the potential impact of interventions to mitigate the effects of malnutrition in communities, through in-depth qualitative research or probabilistic quantitative studies using primary data.

## Resumen

**Objetivo:**

Describir la prevalencia de indicadores de bajo peso y obesidad entre individuos registrados como pueblos y comunidades tradicionales en el Sistema de Vigilancia Alimentaria y Nutricional, en todo Brasil, en 2019.

**Métodos:**

Estudio descriptivo, con datos secundarios individuales de participantes atendidos en atención primaria de Salud del Sistema Único de Salud.

**Resultados:**

En la población estudiada (N = 13.944), hubo mayor prevalencia de talla baja en niños y adolescentes en el sexo masculino (14,2%), cuando se compara con el femenino (11,8%); en la población adulta femenina hubo mayor prevalencia de obesidad (23,0%), en comparación con la masculina (11,3%); se destacó la prevalencia de baja talla para la edad en comunidades ribereñas (18,5%), y obesidad en la población adulta faxinalense (75,1%).

**Conclusiones:**

Las heterogeneidades antropométricas entre comunidades requieren respuestas adaptadas, con énfasis en la atención primaria de salud y en garantizar la seguridad alimentaria y nutricional.

## Resumo

**Objetivo:**

Descrever a prevalência de indicadores de baixo peso e de obesidade entre os indivíduos registrados como povos e comunidades tradicionais no Sistema de Vigilância Alimentar e Nutricional, de todo o Brasil, em 2019.

**Métodos:**

Estudo descritivo, com dados individualizados secundários de participantes atendidos na Atenção Básica do Sistema Único de Saúde.

**Resultados:**

Na população estudada (N = 13.944), houve maior prevalência de altura baixa entre crianças e adolescentes do sexo masculino (14,2%), quando comparados aos do sexo feminino (11,8%); na população adulta do sexo feminino, observou-se maior prevalência de obesidade (23,0%), quando comparada à do sexo masculino (11,3%); destacou-se a prevalência de altura baixa para a idade nas comunidades ribeirinhas (18,5%), e de obesidade na população adulta faxinalense (75,1%).

**Conclusões:**

As heterogeneidades antropométricas entre as comunidades exigem respostas adaptadas, com ênfase na atenção primária à saúde e na garantia da segurança alimentar e nutricional.

## INTRODUCTION

In Brazil, food and nutrition surveillance is a strategy adopted within the scope of the Brazilian National Health System (*Sistema Único de Saúde* - SUS) to monitor and evaluate the nutritional status of the population using Primary Health Care.^1^ This surveillance aims to maintain an up-to-date diagnosis of issues related to food and nutrition, which are relevant to public health, providing support for planning and implementing measures aimed at improving the food and nutrition situation of the Brazilian population.^2^ One of the main tools used to achieve this goal is the Food and Nutrition Surveillance System (*Sistema de Vigilância Alimentar e Nutricional* - SISVAN), where information on food consumption and nutritional status of individuals of both sexes, all age groups and race/skin color, schooling, and those belonging to traditional peoples and communities is recorded.^2^

Traditional peoples and communities are culturally diverse grups that self-identify as such. They have unique forms of social organization, inhabit specific territories and rely on natural resources as a condition for their cultural, social, religious, ancestral and economic reproduction, using knowledge, innovations and practices generated and transmitted through tradition for this purpose.^3^ Despite a legal framework established to ensure their sustainable development, various social vulnerabilities persist in the territories where these peoples live.^4^ These vulnerabilities are attributed to the influence of a modernization paradigm, ethnocentrism, marginalization, and specially economic interests opposed to their *modus vivendi*.^5^ ^),(^ ^6^

Guidelines from the 3^rd^ National Conference on Food and Nutrition Security highlighted the vulnerability of these communities to food insecurity.^7^ This phenomenon can be directly related to the loss of their territories, environmental degradation, climate change, inadequate public policies and social exclusion, which would hinder access to quality and sufficient food, potentially leading to other health problems.^4^ ^),(^ ^6^ ^),(^ ^8^

From an epidemiological perspective, differences have been observed in these communities when compared to the general population. Association between body mass index (BMI) and higher prevalence of hypertension have been found in Quilombola communities, for instance.^9^ Internationally, Roma people living in settlements in Eastern Europe reported a higher prevalence of obesity, metabolic syndrome and parasitic diseases when compared to the non-Roma population.^10^ The other side of malnutrition is also observed in these peoples, as evidenced by high rates of infant mortality and malnutrition in riverine communities in the Amazon.^11^

The anthropometric monitoring of traditional peoples and communities is crucial for planning public policies aimed at promoting health, food and nutrition security. These indicators provide an indirect means to measure the state of food insecurity, guide intervention strategies and monitor the effectiveness of the policies implemented, either in a general or specific context, within each community.^12^

The objective of this study was to describe the prevalence of underweight and obesity indicators among individuals registered as traditional peoples and communities in the SISVAN, Brazil in 2019.

## METHODS

*Study design*

This was a descriptive study, using individual secondary data on the nutritional status of individuals belonging to traditional peoples and communities in Brazil, registered on SISVAN in 2019.

*Setting*

SISVAN is a nationwide health information system that provides continuous data on the food and nutritional status of the population using Primary Health Care within the SUS. Since 2013, the System has provided specific information about traditional people and community.^13^ In 2016, e-SUS Primary Care (e-SUS AB) strategy and SISVAN was integrated, leading to an increase in registrations in the System.^14^

The data for this study were collected in the context of routine visits made by individuals from these communities to public health facilities of Primary Health Care, across the country, as recorded from January 1, 2019 to December 31, 2019.

In 2019, from which data were collected for this study, the Bolsa Família Program provided income transfers to needy families with the condition that they kept their children under 7 years of age in nutritional follow-up.^1^

*Participants*

Data on individuals registered in SISVAN were used, with inclusion criteria based on the record made in 2019 and the individual being recognized as belonging to traditional peoples and communities as indicated in the System’s registration and monitoring form. The year 2019 presented the highest number of records of traditional peoples and communities, and was selected. Other factors influencing the choice of this time period included its immediate proximity to 2020, when physical distancing measures were taken due to the COVID-19 pandemic emergency, and the fact that the Bolsa Família was in force and properly implemented, allowing for comparability with prior years and subsequent years following the resumption of the program. 

The following data were excluded from the study: (i) duplicate observations, keeping the most recent record, (ii) records of adults weighing less than 30 kg and more than 300 kg or with a height greater than 2.20 m and less than 1.20 m, as recommended by the Ministry of Health for the Chronic Disease Risk and Protective Factors Surveillance Telephone Survey,^15^ and (iii) observations of children or adolescents with Z-scores, considered implausible according to the WHO recommendation for children’s anthropometric surveys.^16^

*Variables*

The variables used were classified into two groups: sociodemographic and anthropometric.

Sociodemographic variables included:

a) national macro-region of residence (North; Northeast; Southeast; South; Midwest);

b) sex (female; male);

c) age group, derived from time difference between the date of follow-up and the date of birth (0 to 5 months; 6 to 11 months; 1 year; 2 years; 3 to 5 years; 6 to 10 years; 11 to 19 years; 20 to 39 years; 40 to 49 years; 50 to 59 years; 60 to 69 years; 70 to 79 years; 80 years and older);

d) race/skin color (White, mixed-race, Black, Indigenous and Asian);

e) schooling (incomplete elementary education or less; complete elementary education; complete high school; complete higher education or more);

f) traditional people or communities, as per SISVAN registration and monitoring form (Quilombola communities; Agro-extractivist communities; *Caatingueiros*; Caiçaras; *Fundo e fecho de pasto* communities; Cerrado communities; Extrativist communities; Faxinalense communities; *Geraizeiros*; Shellfish gatherers; Pantanal dwellers; *Artisanal* fishermen; Pomeranians; Roma people; *Terreiro* communities; Babassu coconut breakers; *Retireiros*; Riverine dwellers; Rubber tappers; *Vazanteiros*).^17^

The anthropometric variables were:

a) weight, in kilograms (kg);

b) height or length, in centimeters (cm);

c) weight-for-age Z-scores;^18^

d) height-for-age Z-scores;^18^

e) BMI-for-age Z-scores;^18^

f) BMI, in kilograms per height in metres squared (kg/m²);^18^

g) indicator of low weight-for-age in children (Z-score < -2);^19^

h) indicator of low height-for-age in children and adolescents (Z-score < -2);^19^

i) indicator of low weight and height-for-age, using the adapted Waterlow criterion, in children (Z-scores < -2, for both);^19^

j) indicator of high weight-for-age in children (Z-score > 2);^19^

k) underweight in adults (BMI < 18.5 kg/m²) and in the elderly (BMI ≤ 22 kg/m²);^18^

l) obesity in adults (BMI ≥ 30 kg/m²);^18^

m) overweight in adults (BMI ≥ 25 kg/m²) and in the elderly (BMI ≥ 27 kg/m²).^18^

*Data Sources and Measurement*

The individual data from SISVAN were obtained through an official letter, in which data access was requested from the Secretariat of Primary Health Care, accompanied by a duly completed Responsibility Agreement and the documentation listed in the specific ordinance.^20^

Regarding data measurement, it is worth noting that there are protocols for collecting anthropometric data within the scope of SISVAN, covering all stages of life. However, it is not possible to ensure that all professionals responsible for the data measurement used in this study had access to calibrated instruments or underwent standardized training, given the breadth and heterogeneity of the Brazilian reality. For children up to 2 years of age, it is recommended that weight be collected on pediatric scales, whether mechanical or digital, positioning the child to evenly distribute the weight. Length should be measured using infantometers, with the child’s head firmly placed against the fixed part of the equipment, the neck straight and the chin away from the chest, aligned with the Frankfurt plane.^18^

For people over 2 years of age, it is important to ensure that weight is measured by means of mechanical platform scales or digital scales. The individual should be positioned with his or her back to the scale, barefoot, wearing little clothing, in the center of the equipment, standing upright, with feet together and the arms extended alongside the body. For height measurement a stadiometer is recommended. The individual should stand barefoot and without any head accessories, centered on the device, standing upright, with arms extended alongside the body, looking straight ahead at eye level, with their head raised, and aligned with the Frankfurt plane.^18^

*Bias*

In order to reduce the risk of bias, SISVAN protocols^2^ ^),(^ ^17^ ^),(^ ^18^ ^),(^ ^21^were observed ensuring consistency between routine procedures and the analysis performed. In addition to these measures, the exclusion criterion for implausible anthropometric values was applied to reduce possible errors in weight and height measurements, or in the digitization of information in the System.

*Statistical methods*

Data quality was investigated and no evidence of inconsistencies was identified, as recommended by the WHO.^16^ The graphic material related to this verification is available in supplementary material. The distribution of absolute and relative frequencies of sociodemographic and anthropometric characteristics was described by sex. Bivariate quantile regressions were conducted, with “weight”, “height” or “BMI” as the dependent variable; and “age” as the independent variable. Quadratic terms were included in all models, accounting for curvilinear relationships between each of the independent variables and the dependent variable. Each bivariate regression was performed five times for the percentiles 3, 15, 50, 85, and 97. Scatterplots were created, showing the observed points and the estimated curves in the quantile regressions for each percentile mentioned.

Z-scores were calculated based on weight, height, age, and gender data, according to the growth curves provided by the WHO.^22^ The mean and 95% confidence interval (95%CI) of BMI and weight-for-age, height-for-age, and BMI-for-age Z-scores were calculated according to each traditional people and community. Finally, the prevalence and its respective 95%CI were described for the following indicators: low height-for-age in children and adolescents; low weight-for-age, low weight and height-for-age, and overweight in children; and underweight, obesity and overweight in adults.

The manipulation of the database related to the total population registered on SISVAN was performed using the Google Colab application for the Python programming language. After selection, the data were stored, cleaned and analyzed using the Stata software, version 16. Missing values have been handled with row exclusion.

*Ethical aspects*

Anonymous individual information was provided by the Secretariat of Primary Health Care of the Ministry of Health (*Secretaria de Atenção Primária à Saúde, do Ministério da Saúde* - SAPS/MS) and was exclusively used for statistical purposes. In obtaining the data, the social relevance of the study and the assessment of potential risks and benefits were taken into account, as recommended by the National Health Council (*Conselho Nacional de Saúde* - CNS). The study project was approved by the Research Ethics Committee of the Universidade de Fortaleza (Coética/Unifor): with the approval process initiated in April 2020, under Opinion No. 4,348,452 and Certificate of Submission for Ethical Appraisal (CAAE) No. 31540320.9.1001.5052.

## RESULTS

**Figure 1:**
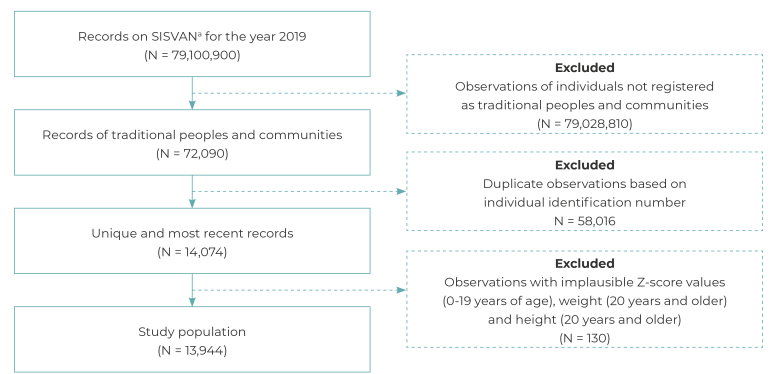
Selection of the study population

The study population was comprised of 13,944 individuals of both sexes, aged between 0 and 101 years. This sample size resulted from the application of inclusion and exclusion criteria based on 79,100,900 records from 2019, nationwide (Figure 1).

a) SISVAN: *Sistema de Vigilância Alimentar e Nutricional* (Food and Nutrition Surveillance System).

Table 1 describes the sociodemographic and anthropometric characteristics of the study population, stratified by sex. The most reported traditional peoples and communities were riverine dwellers (50.3%), geraizeiros (13.3%) and quilombolas (9.9%). The communities with the smallest population registered on SISVAN were the babassu coconut breakers (< 0.1%), Faxinalenses (0.1%) and the Roma people (0.1%).

**Table 1:** Sociodemographic and anthropometric characteristics of Brazilians belonging to traditional peoples and communities registered on SISVAN, by sex (N = 13,944), Brazil, 2019

Characteristics	Female			Male		
	N	%	(95%CI^a^)	N	%	(95%CI^a^)
Macro-region (n = 13,944)						
North	5,092	57.3	(56.2;58.3)	2,779	55.0	(53.6;56.4)
Northeast	1,965	22.1	(21.3;23.0)	937	18.5	(17.5;19.6)
Southeast	1,522	17.1	(16.4;17.9)	1,115	22.1	(20.9;23.2)
South	163	1.8	(1.6;2.1)	144	2.8	(2.4;3.3)
Midwest	148	1.7	(1.4;2.0)	79	1.6	(1.3;1.9)
Age group, in complete years (n = 13,944)						
< 5	943	10.6	(10.0;11.3)	973	19.3	(18.2;20.4)
5-9	1,651	18.6	(17.8;19.4)	1,428	28.3	(27.0;29.5)
10-19	2,083	23.4	(22.6;24.3)	816	16.1	(15.2;17.2)
20-29	1,435	16.1	(15.4;16.9)	375	7.4	(6.7;8.2)
30-39	1,054	11.9	(11.2;12.5)	343	6.8	(6.1;7.5)
40-49	621	7.0	(6.5;7.5)	307	6.1	(5.4;6.8)
50-59	434	4.9	(4.5;5.3)	299	5.9	(5.3;6.6)
60-69	367	4.1	(3.7;4.6)	302	6.0	(5.4;6.7)
70-79	212	2.4	(2.1;2.7)	148	2.9	(2.5;3.4)
≥ 80	90	1.0	(0.8;1.2)	63	1.2	(1.0;1.6)
Race/skin color (n = 13,944)						
Mixed-race	5,844	65.7	(64.7;66.7)	3,218	63.7	(62.3;65.0)
White	1,151	12.9	(12.3;13.7)	762	15.1	(14.1;16.1)
Asian	655	7.4	(6.8;7.9)	337	6.7	(6.0;7.4)
Black	445	5.0	(4.6;5.5)	249	4.9	(4.4;5.6)
Indigenous	369	4.2	(3.8;4.6)	225	4.5	(3.9;5.1)
Without information	426	4.8	(4.4;5.3)	263	5.2	(4.6;5.9)
Schooling, by level of education (n = 13,944)						
Incomplete elementary education or less	4,940	55.6	(54.5;56.6)	3,189	63.1	(61.8;64.4)
Complete elementary education	1,791	20.1	(19.3;21.0)	715	14.1	(13.2;15.1)
Complete high school	706	7.9	(7.4;8.5)	271	5.4	(4.8;6.0)
Complete higher education	66	0.7	(0.6;0.9)	16	0.3	(0.2;0.5)
Without information	1,387	15.6	(14.9;16.4)	863	17.1	(16.1;18.1)
System of origin (n = 13,944)						
e-SUS AB	4,466	50.2	(49.2;51.3)	3,443	68.1	(66.8;69.4)
Programa Bolsa Família	3,329	37.4	(36.4;38.5)	641	12.7	(11.8;13.6)
SISVAN^b^	1,095	12.3	(11.7;13.0)	970	19.2	(18.1;20.3)
Traditional peoples or communities (n = 13,944)						
Riverine dwellers	4,463	50.2	(49.2;51.2)	2,557	50.6	(49.2;52.0)
Geraizeiros	1,181	13.3	(12.6;14.0)	671	13.3	(12.4;14.2)
Quilombolas	883	9.9	(9.3;10.6)	503	10.0	(9.2;10.8)
Caiçaras	349	3.9	(3.5;4.4)	314	6.2	(5.6;6.9)
Vazanteiros	298	3.4	(3.0;3.7)	245	4.8	(4.3;5.5)
Caatingueiros	318	3.6	(3.2;4.0)	173	3.4	(3.0;4.0)
Agro-extrativist communities	330	3.7	(3.3;4.1)	143	2.8	(2.4;3.3)
Artisanal fishermen	212	2.4	(2.1;2.7)	56	1.1	(0.9;1.4)
Cerrado Communities	145	1.6	(1.4;1.9)	62	1.2	(1.0;1.6)
Extrativist communities	169	1.9	(1.6;2.2)	29	0.6	(0.4;0.8)
Pantanal dwellers	110	1.2	(1.0;1.5)	33	0.7	(0.5;0.9)
Shellfish gatherers	83	0.9	(0.8;1.2)	44	0.9	(0.6;1.2)
Retireiros	80	0.9	(0.7;1.1)	41	0.8	(0.6;1.1)
Fundo e fecho de pasto communities	77	0.9	(0.7;1.1)	38	0.8	(0.5;1.0)
Pomeranians	65	0.7	(0.6;0.9)	43	0.9	(0.6;1.1)
Terreiro communities	51	0.6	(0.4;0.8)	46	0.9	(0.7;1.2)
Rubber tappers	55	0.6	(0.5;0.8)	37	0.7	(0.5;1.0)
Roma people	9	0.1	(0.1;0.2)	8	0.2	(0.1;0.3)
Faxinalenses	8	0.1	(0.0;0.2)	8	0.2	(0.1;0.3)
Babassu coconut breakers	4	0.0	(0.0;0.1)	3	0.1	(0.0;0.2)
Indicator of low height-for-age (< 20 years) (n = 7,649)						
No	3,944	88.2	(87.2;89.1)	2,725	85.8	(84.5;87.0)
Yes	529	11.8	(10.9;12.8)	451	14.2	(13.0;15.5)
Indicator of low weight-for-age (< 5 years) (n = 1,916)						
No	908	96.3	(94.9;97.3)	941	96.7	(95.4;97.7)
Yes	35	3.7	(2.7;5.1)	32	3.3	(2.3;4.6)
Indicator of low weight and height-for-age (< 5 years) (n = 1,916)						
No	924	98.0	(96.9;98.7)	952	97.8	(96.7;98.6)
Yes	19	2.0	(1.3;3.1)	21	2.2	(1.4;3.3)
Indicator of overweight (< 5 years) (n = 1,916)						
No	892	94.6	(93.0;95.9)	927	95.3	(93.7;96.4)
Yes	51	5.4	(4.1;7.0)	46	4.7	(3.6;6.3)
Underweight (≥ 20 years) (n = 6,050)						
No	3,996	94.8	(94.1;95.5)	1,732	94.3	(93.1;95.3)
Yes	217	5.2	(4.5;5.9)	105	5.7	(4.7;6.9)
Obesity (≥ 20 and ≤ 59 years) (n = 4,868)						
No	2,729	77.0	(75.6;78.4)	1,174	88.7	(86.8;90.3)
Yes	815	23.0	(21.6;24.4)	150	11.3	(9.7;13.2)
Overweight (≥ 20 years) (n = 6,050)						
No	1,852	44.0	(42.5;45.5)	1,059	56.0	(55.4;59.9)
Yes	2,361	57.6	(54.5;57.5)	778	42.4	(40.1;44.6)

a) 95%CI: 95% confidence interval b) SISVAN: *Sistema de Vigilância Alimentar e Nutricional* (Food and Nutrition Surveillance System).

There was a predominance of participants from the North region (56.4%), followed by the Northeast (20.8%), Southeast (18.9%), South (2.2%) and Midwest (1.6%) regions. There was a higher frequency in the age group of 10 to 19 years in females (23.4%), while in males, it was from 5 to 9 years old (28.3%). The most common race/skin color was mixed-race (65.0%), followed by White (13.7%), Asian (7.1%), Black (5.0%) and Indigenous (4.3%). The most frequent education level was “incomplete elementary education or less” (58.3%), and the majority of records were made on the e-SUS AB system (56.7%) (Table 1).

There was a higher prevalence of indicators of low height-for-age among male children and adolescents (14.2%), when compared to the same indicators in females (11.8%). There was no evidence of a higher prevalence of indicators of low weight-for-age and high weight-for-age among children and adolescents of either sex. Regarding records of the adult female population, there was a higher prevalence of obesity (23.0%) and overweight (57.6%) when compared to males (11.3% and 42.4%, respectively) (Table 1).

**Figure 2:**
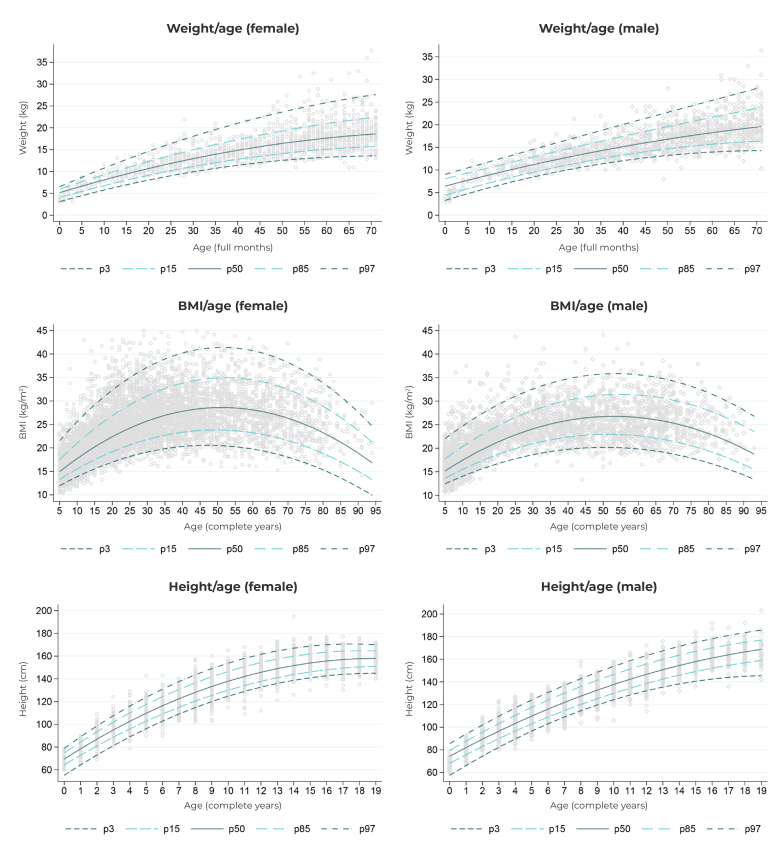
- Distribution and percentiles of weight, height and body mass index of Brazilians belonging to traditional peoples and communities registered on SISVAN,^a^ according to sex and age, Brazil, 2019

The distribution of weight by age according to sex is presented in Figure 2. It could be seen that the median weight, height and BMI of the individuals did not show a linear relationship with age, but rather a curvilinear one. Observations regarding weight in children were similarly distributed between sexes, being slightly higher for males at the age of five. The distribution of height in the population aged 0 to 19 years differed between the sexes, becoming more evident from the age of 14, when the median for females tended to stabilize, compared to the median observed for males, which continued to increase until the age of 19. In addition, a higher median BMI was observed in the age group of 50 to 55 years for both sexes.

a) SISVAN: Sistema de Vigilância Alimentar e Nutricional (Food and Nutrition Surveillance System).

Note: Lines estimated from quantile regression models related to the 3rd, 15th, 50th, 85th and 97th percentiles, with anthropometric measurement as the dependent variable and age as the independent variable. Quadratic terms were included in the model to account for curvilinear relationships.

**Figure 3:**
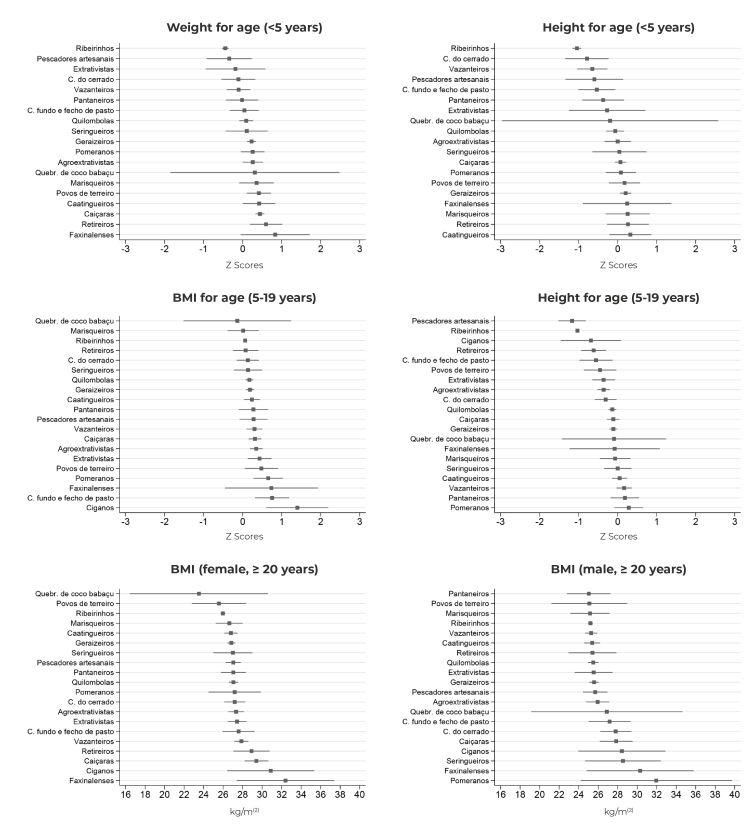
Mean and 95% confidence intervals of Z-scores and body mass index of Brazilians belonging to traditional peoples and communities registered on SISVAN,^a^ according to age group and belonging, Brazil, 2019

Legend: ribeirinhos (Riverine dwellers); pescadores artesanais (Artisanal fishermen); extrativistas (Extractivist communities); comunidades do cerrado (Cerrado communities); pantaneiros (Pantanal dwellers); comunidades fundo e fecho de pasto (Fundo e fecho de pasto communities); seringueiros (Rubber tappers); pomeranos (Pomeranians); agroextrativistas (Agro-extractivist communities); Quebradeiras de coco de babaçu (Babassu coconut breakers); marisqueiros (Shellfish gatherers); and povos de terreiro (Terreiro communities).

The communities with the lowest mean weight-for-age and height-for-age Z-scores were traditional riverine dwellers. Babassu coconut breaker communities had the lowest mean BMI-for-age and BMI Z-scores in adult females. Roma people showed the highest mean weight-for-age Z-scores up to 5 years of age, while the Faxinalense communities reported the highest mean weight-for-age Z-scores in children and BMI in females. The highest mean BMI in males occurred in the Pomeranian communities (Figure 3).

**Figure 4:**
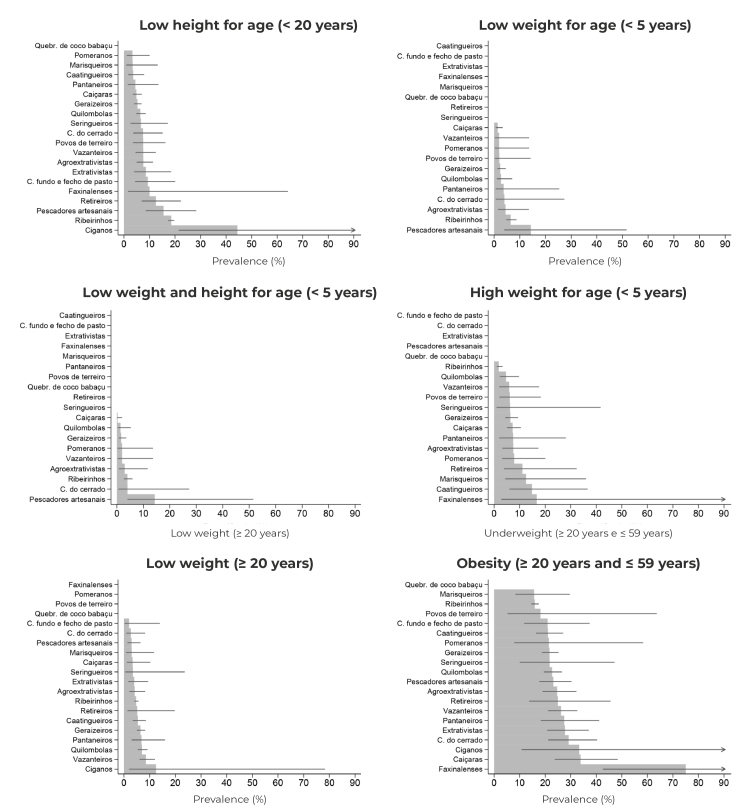
Prevalence of indicators of underweight and obesity in Brazilians belonging to traditional peoples and communities registered on SISVAN, according to age group and belonging group, Brazil, 2019

Legend: ribeirinhos (Riverine dwellers); pescadores artesanais (Artisanal fishermen); extrativistas (Extractivist communities); comunidades do cerrado (Cerrado communities); pantaneiros (Pantanal dwellers); comunidades fundo e fecho de pasto (Fundo e fecho de pasto communities); seringueiros (Rubber tappers); pomeranos (Pomeranians); agroextrativistas (Agro-extractivist communities); Quebradeiras de coco de babaçu (Babassu coconut breakers); marisqueiros (Shellfish gatherers); and povos de terreiro (Terreiro communities).

Prevalence of anthropometric indicators is presented in Figure 4 and detailed in the supplementary material. The prevalence of low height-for-age was higher in Roma communities (44.4%). In riverine communities, there was a higher prevalence of low height-for-age (18.5%), which was statistically significant when compared to the prevalence in other communities - with the exception of *retireiro*, artisanal fisherman, extrativist, *fundo e fecho de pasto* and Faxinalense communities. Among the traditional peoples and communities in which there were cases indicative of low weight-for-age, riverine communities showed a higher prevalence of this indicator (6.5%), when compared to the same prevalence among Caiçara (1.4%) and *Geraizeiro* (2.4%) communities. Faxinalense communities showed a higher prevalence of obesity (75.1%), when compared to the same prevalence in other traditional peoples and communities - with the exception of terreiro, Pomeranian, rubber tapper, *Retireiro*, Roma and *Caiçara* communities.

## DISCUSSION

Higher prevalence of low weight-for-age in children was observed in riverine dwellers, when compared to other traditional peoples and communities. Riverine dwellers and artisanal fisherman communities also showed the highest prevalence of low height-for-age among children and adolescents. Faxinalense communities presented the highest prevalence of obesity among adults, as well as overweight, when compared to other communities studied.

The most prevalent traditional people or community were the riverine dwellers, who showed the second-highest prevalence of low height-for-age and low weight-for-age indicators. These communities are predominantly located in the Amazon territory, and their health indicators have been often described in international reports.^11^ The environmental challenges faced in these territories, with intermittent cycles of droughts and floods, result in seasonal difficulties in fishing activities,^23^ in addition to the factors observed in federal governance, such as the extinction of the National Council for Food and Nutrition Security in 2019.^24^ The failure to meet targets for food and nutrition security projects for Amazonian populations in 2019^25^ and a focus on emergency food security actions, rather than structural measures for indigenous populations,^26^ may have led to situations of food insecurity, manifested by insufficient food, and influenced the high prevalence of underweight in children found in this study.

The majority of traditional peoples or communities in this study showed indicators of low weight-for-age or low height-for-age in children or adolescents. According to the criteria proposed by the WHO,^16^ the prevalence of low weight-for-age is considered high at the threshold of 10%, a figure that artisanal fishing communities exceeded by 43.0% (14.3%). In the coastal area of Salvador, the capital city of the state of Bahia, the precariousness of the fishing sector was investigated in artisanal fishing and shellfish gatherer communities in 2020, revealing a lower level of food security and social support, as well as diminished expectations regarding receiving welfare benefits and collaborating with other sectors of society.^27^

Roma, Caiçara and Faxinalense communities showed the highest percentages of obesity among adults. Issues related to a high prevalence of obesity in Roma people were observed in Slovakia, where the prevalence of obesity in adults was 29% higher when compared to that of the general adult population

This phenomenon was explained by eating habits related to increased caloric intake, in a context of limited access to health services.^10^ With regard to the Caiçara communities, representatives of these communities report the influence of real estate speculation on their way of life and in their territories, leading to losses of their traditions, and population exodus.^28^ Such factors may contribute to the consumption of highly processed foods, given the increased availability of these products in urban food environments.^29^

At the international level, the Brazilian concept of traditional peoples and communities^3^ finds a semantic parallel in the expression “peasants and other people working in rural areas”, as applied in the United Nations Declaration on the Rights of Peasants and Other People Working in Rural Areas.^30^ This document also raises concern about the disproportionate suffering of these peoples from poverty, hunger and malnutrition; in this sense, the Declaration proposes measures to ensure the right of these peoples to food and food sovereignty, emphasizing the fight against child malnutrition, the determination of their food systems, and the restriction on the use of agricultural products harmful to health.

The limitations of this study can be categorized into two groups: the generalization of the results beyond the respective study populations; and the degree of precision of the anthropometric indicators used. Given the possibility of a higher number of individuals seeking welfare benefits in this population, and taking into consideration the heterogeneities among the Brazilian regions with regard to the human and material resources and use of healthcare services, generalizations of the results of this study should not be extended to individuals belonging to traditional peoples and communities who are not recorded in SISVAN. It is worth highlighting the nature of the indicators used, which approximate complex phenomena related to malnutrition and overweight, focused on the population thinking and, therefore, should be understood with the necessary clinical caveats, incorporating anthropological and genetic insights.

When describing the distribution of anthropometric indicators and the prevalence of underweight and obesity, the importance of the SISVAN for monitoring the nutritional status of traditional peoples and communities, some of the most vulnerable groups to food insecurity in Brazil, is reaffirmed. The findings highlight significant differences in nutritional status indicators among the communities observed, revealing the need for targeted research, within the context of each community, supporting interventions aimed at reducing or eliminating inequities experienced in the field of food and nutrition.

## Figures and Tables

**Supplementary figure 1:**
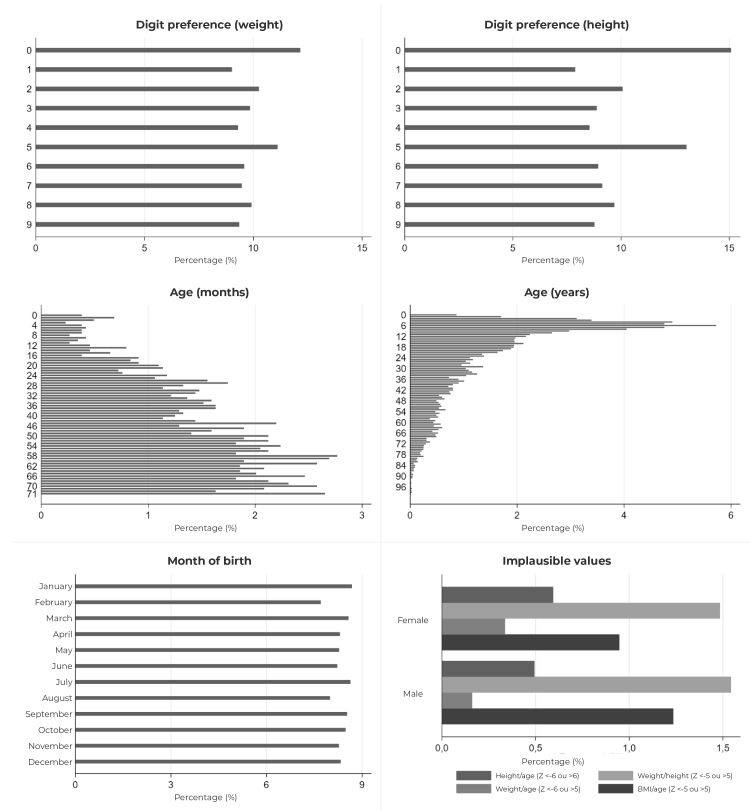
- Analysis of data quality for Brazilians belonging to traditional peoples and communities registered in SISVAN, Brazil, 2019

**Supplementary figure 2:**
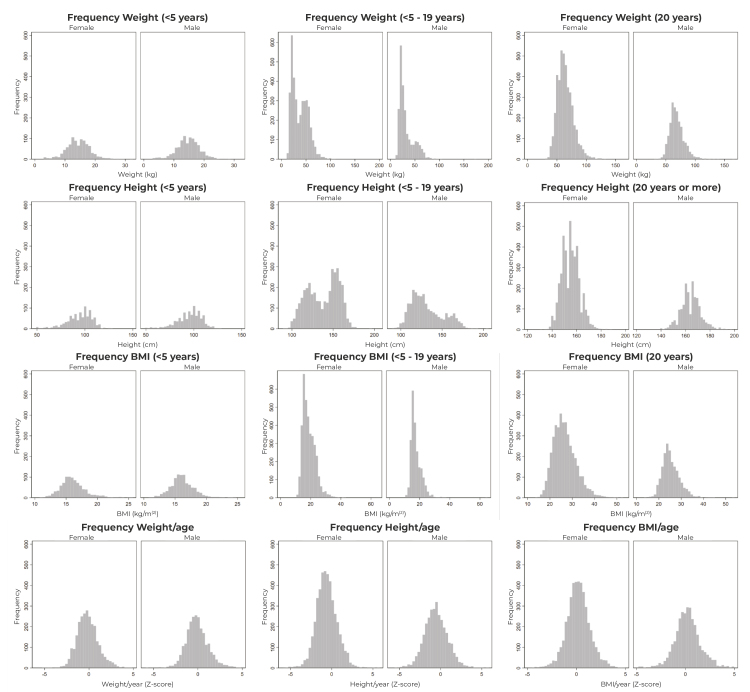
- Frequency distribution of weight, height, BMI and Z-scores of Brazilians belonging to traditional peoples and communities registered in SISVAN, Brazil, 2019

## Supplementary figure 1

### - Analysis of data quality for Brazilians belonging to traditional peoples and communities registered in SISVAN, Brazil, 2019

**Supplementary table 1:** - Prevalence of anthropometric indicators of Brazilians belonging to traditional peoples and communities registered in SISVAN, according to group and age (n = 13,944), Brazil, 2019

Traditional peoples or communities	< 20 years	< 5 years		≥ 20 years		≥ 20 and ≤ 59 years	
	Chronic malnutrition	Acute malnutrition	Chronic decompensated malnutrition	Overweight	BUnderweight	Overweight	Obesity
	n (%)^a^	n (%)^b^	n (%)^c^	n (%)^d^	n (%)^e^	n (%)^f^	n (%)^g^
Riverine dwellers	758 (18.5)	39 (6.5)	24 (4.0)	11 (1.8)	132 (4.8)	1,323 (47.7)	383 (16.0)
Geraizeiros	48 (5.3)	9 (2.4)	6 (1.6)	24 (6.4)	58 (6.4)	479 (53.2)	133 (21.7)
Quilombolas	44 (6.4)	4 (2.7)	2 (1.3)	7 (4.7)	48 (7.0)	370 (54.3)	123 (22.7)
Caiçaras	28 (4.9)	5 (1.4)	1 (0.3)	26 (7.2)	3 (3.4)	57 (64.0)	20 (33.9)
Vazanteiros	14 (7.5)	1 (2.0)	1 (2.0)	3 (5.9)	30 (8.5)	184 (52.4)	60 (26.2)
Caatingueiros	6 (3.6)	0 (0.0)	0 (0.0)	4 (14.8)	17 (5.4)	164 (51.9)	48 (21.1)
Agro-extractivist communities	21 (7.6)	3 (4.5)	2 (3.0)	5 (7.5)	8 (4.2)	107 (56.0)	41 (24.7)
Artisanal fishermen	9 (15.5)	2 (14.3)	2 (14.3)	0 (0.0)	6 (2.9)	125 (61.0)	41 (23.2)
Cerrado communities	7 (7.4)	1 (4.0)	1 (4.0)	0 (0.0)	3 (2.7)	68 (61.3)	26 (29.2)
extractivist communities	6 (8.6)	0 (0.0)	0 (0.0)	0 (0.0)	5 (4.0)	78 (62.4)	33 (27.7)
Pantanal dwellers	3 (4.5)	1 (3.7)	0 (0.0)	2 (7.4)	5 (6.8)	39 (53.4)	17 (27.4)
Shellfish gatherers	2 (3.4)	0 (0.0)	0 (0.0)	3 (12.5)	2 (3.0)	41 (61.2)	8 (15.7)
Retireiros	10 (12.5)	0 (0.0)	0 (0.0)	3 (11.1)	2 (5.1)	25 (64.1)	8 (25.0)
Fundo e fecho de pasto communities	6 (9.4)	0 (0.0)	0 (0.0)	0 (0.0)	1 (2.0)	31 (62.0)	9 (20.9)
Pomeranians	3 (3.3)	1 (2.0)	1 (2.0)	4 (7.8)	0 (0.0)	12 (80.0)	3 (21.4)
Terreiro communities	6 (7.5)	1 (2.0)	0 (0.0)	3 (6.1)	0 (0.0)	7 (41.2)	2 (18.2)
Rubber tappers	4 (6.7)	0 (0.0)	0 (0.0)	1 (6.3)	1 (3.4)	19 (65.5)	5 (21.7)
Roma people	4 (44.4)	0 (0.0)	0 (0.0)	0 (0.0)	1 (12.5)	5 (62.5)	2 (33.3)
Faxinalenses	1 (10.0)	0 (0.0)	0 (0.0)	1 (16.7)	0 (0.0)	5 (83.3)	3 (75.0)
Babassu coconut breakers	0 (0.0)	0 (0.0)	0 (0.0)	0 (0.0)	0 (0.0)	0 (0.0)	0 (0.0)
Total	980 (12.8)	67 (3.5)	40 (2.1)	97 (5.1)	322 (5.3)	3,139 (51.9)	965 (19.8)

a) Count (n) and prevalence proportion (%) of cases indicative of chronic malnutrition (height-for-age Z score < -2) among individuals in the respective belonging group aged less than 20 years. b) Count (n) and prevalence proportion (%) of cases indicative of acute malnutrition (weight-for-age Z score < -2) among individuals in the respective belonging group aged less than 5 years. c) Count (n) and prevalence proportion (%) of cases indicative of chronic decompensated malnutrition (height-for-age Z score < -2 together with weight-for-age Z score < -2) among individuals in the respective belonging group aged less than 5 years. d) Count (n) and prevalence proportion (%) of cases indicating overweight (weight-for-age Z score > 2) among individuals in the respective belonging group aged less than 5 years. e) Count (n) and prevalence proportion (%) of cases indicating underweight (BMI < 18.5 kg/m² for adults and ≤ 22.0 kg/m² for the elderly) among individuals in the respective belonging group aged 20 years or older. f) Count (n) and prevalence proportion (%) of cases indicating overweight (BMI ≥ 25.0 kg/m² for adults and ≥ 27.0 kg/m² for the elderly) among individuals in the respective belonging group aged 20 years or older. g) Count (n) and prevalence proportion (%) of cases indicative of obesity (BMI ≥ 30.0 kg/m²) among individuals in the respective group aged 20 years or more and 59 years or less.
